# Impact of settings and culture on nurses’ knowledge of and attitudes and perceptions towards people with dementia: An integrative literature review

**DOI:** 10.1002/nop2.1106

**Published:** 2021-10-30

**Authors:** Sara Mahmoud Yaghmour

**Affiliations:** ^1^ University of Southampton Southampton UK; ^2^ King Abdulaziz University Jeddah Saudi Arabia

**Keywords:** acute care in dementia, attitudes, Dementia, integrative review, knowledge, neurocognitive disorders, nursing care, nursing homes, perceptions

## Abstract

**Background:**

Well‐trained nurses are required to support dementia patients; however, the quality of the provided dementia nursing care can be impacted by nurses’ knowledge, attitudes and perceptions towards people with dementia.

**Aim:**

To obtain an overview of the current nursing practice towards people with dementia around the world.

**Design:**

An integrated literature review was conducted based on Whittemore and Knafl's method.

**Results:**

A total of 72 articles met the inclusion criteria. Three main themes were identified: (1) nurses’ knowledge, attitudes and perceptions towards dementia; (2) nursing experience of caring for people with dementia in acute and community care settings; and (3) dementia nursing care across health regions.

**Conclusion:**

Nurses play the role of facilitators in the efficient delivery of quality care for dementia patients. A variety of attitudes and perceptions towards people with dementia were found to be triggered by the severity of dementia, religion, ethnicity and gender.

**Implications for practice:**

Healthcare organisations and educational settings need to coordinate and function together to improve nurses’ knowledge and encourage positive attitudes towards people with dementia.

**What does this research add to the existing knowledge in gerontology?**

- This review examined the different strategies used by nurses in caring for dementia patients and the measures that can be taken to improve their knowledge, attitudes and perceptions in both acute hospital and community care settings.
- Nurses’ burnout, ward routine and work environment were found to have a negative impact on nurses’ perceptions towards dementia patients.
- Nurses’ perceptions towards end‐of‐life and holistic care are influenced by the religious and cultural practices of the person with dementia.

**What are the implications of this new knowledge for nursing care with older people?**

- It is suggested to include both theoretical and practical interventions to enhance nurses’ knowledge and attitudes towards people with dementia.
- Nurses in administrative roles must acquire knowledge about skills to care for people with dementia, and they must support nurses in providing advanced and high‐quality care.
- Communication competencies and strategies to care for dementia patients have to be defined and added to core nursing competencies for dementia care.
- Nurses must be aware of people with dementia actual condition and have the proper knowledge and skill set for managing people with dementia and their specific needs.

**How could the findings be used to influence policy, practice, research or education?**

- Healthcare organisations and educational settings should work together to enhance nurses’ knowledge and encourage positive attitudes towards people with dementia.
- Furthermore, studies adapting mixed methodologies are needed to validate the best practices and generalize the findings in the context of nursing care for dementia across the world.
- The policies and practices within community care centres and acute care hospital settings require improvement to maintain patients’ expectations about care.

## 1 BACKGROUND

Dementia is one of the most prevalent health‐related conditions across the globe, and it is estimated that around 35.6 million people are living with dementia worldwide (American Psychiatric Association, 2012; Briggs et al., 2017). Dementia is one of the most prevalent psychiatric conditions that affects the ageing population, mostly adults above the age of 65 (Prince et al., 2015). Dementia causes multiple symptoms that are characterized by memory impairment, language problems, motor speech disorder, sensory recognition problems and general body functioning disturbance (Prince et al., 2015). The nature of the disease dictates that advanced nursing care must be provided for the successful management of dementia. People with dementia need to receive care from well‐trained nurses in both acute hospital and community care settings so that patients can receive around‐the‐clock care (Strøm et al., 2019).

Despite the fact that nurses play a special role in attending to the special needs of dementia patients, in many cases, it has been found that dementia patients do not receive optimal care (Registered Nurses’ Association of Ontario, 2018). It has been suggested that the provision of care for dementia patients extends beyond the confines of nursing education and overlaps with additional factors, such as attitudes and perceptions about this illness (Daniel et al., 2014). In 2019, authors reported that nursing students have almost no knowledge about how to provide ideal care to patients with dementia; they often struggle in dealing with the behavioural problems of patients (Strøm et al., 2019). Thus, because knowledge and attitude influence the quality of care provided, it is important to distinguish between nursing knowledge, attitudes and perceptions when considering the educational skills of nurses. Each of these three concepts has a different meaning and function. For example, knowledge is associated with cognitive expertise derived from learning, which outlines the role of modern nurses in health care (Hatamleh & Sorio, 2017). The attitude of a nursing professional relates to the individual's belief system, which may or may not be the same as widely accepted societal attitudes (Coban et al., 2015; Yaghmour et al., 2019). In addition, it is well known that societal attitudes also vary depending on the country and culture. Perceptions, on the other hand, are associated with the views, concerns and interpretation of behaviours.

It has been noted that nursing care for people with dementia is different between organisations and it varies according to nursing experience, wards and its business, nurses’ knowledge and their perceptions about the disease, and their attitudes towards people with dementia and dementia care (de Witt & Ploeg, 2016; Eritz et al., 2016; Schindel Martin et al., 2016; Yaghmour et al., 2019; Yaghmour & Gholizadeh, 2016). Therefore, exploring the current nursing practices for people with dementia from a global perspective was significant in order to provide policymakers educational organisations and healthcare organisations with the best available evidence.

The main objective of the current paper was to carry out an integrated literature review of nurses’ knowledge, attitudes and perceptions towards caring for dementia patients in different care settings, including acute care hospitals and community care settings. This is to obtain an overview of the current nursing practice towards people with dementia around the world by exploring the cultural difference impact on nurses’ knowledge, attitudes and perceptions. This will help in identifying any existing gaps in knowledge and opportunities for nursing practice development in the field of mental health. This could pave the way for new reforms in mental health nursing and geriatric educational programmes.

## 2 METHODS

This integrative review was guided by Whittemore and Knafl's method. This method's guidelines were used to assist the data extract and analysis of the included papers (Whittemore & Knafl, 2005). An integrative method involves the inclusion of both empirical and theoretical publications. This methodological approach consists of five stages to guide the review. This includes problem identification, literature search, data evaluation, data analysis and presentation. This section provides comprehensive details of stages two and three, which relate to the description of a comprehensive search strategy, explaining the methodological quality and representativeness of the primary studies (Hopia et al., 2016).

### 2.1 Search strategy

A systematic search was conducted in March 2018 and updated in June 2021 using a number of scientific databases, including DelphiS, CINAHL, MEDLINE, OVID, ProQuest, EMBASE and PsychINFO, using keywords such as “nurses,” “staffing,” “knowledge,” “attitude,” “perception” and “dementia.” Several truncations were used to further refine the key terms and ensure that all relevant articles related to the research question were taken. The key truncations used included “Nurs* staff” OR “register* nurse*” in combination with knowledge, educational, understand*, awareness, attitude, perception, opinion, thought, feeling, OR beliefs. Search terms like dementia, Alzheimer*, “Lewy body,” Parkinson*, “mild cognitive impairment,” “cognitive impairment,” “cognitive decline,” “memory loss,” “cognitive function,” OR “cognitive dysfunction” were also used during the search process (Table 1).

**TABLE 1:** Search terms and alternative terms/synonyms

Main term	Nurse		Learning		Attitude		Perception		Dementia
Alternate terms	‘Nurs* staff’ OR ‘register* nurse*’	AND	knowledge OR educational OR understand* OR awareness	AND	attitude OR thought OR feeling	AND	perception OR opinion OR Beliefs	AND	dementia OR Alzheimer* OR ‘Lewy body’ OR Parkinson* OR ‘mild cognitive impairment’ OR ‘cognitive impairment’ OR ‘cognitive decline’ OR ‘memory loss’ OR ‘cognitive function’ OR ‘cognitive dysfunction’

### 2.2 Eligibility criteria

The inclusion criteria focused on defining the target sample group who were Registered Nurses, research design, publication date, language and main outcome in the articles selected for review. A summary of the inclusion/exclusion criteria is presented in Table 2. The exclusion criteria for article selection were also defined. All nursing students were excluded from the review. In addition, studies investigating patients with mental disorders other than dementia were excluded.

**TABLE 2:** Inclusion and exclusion criteria

Inclusion/exclusion criteria		Justification
Study participants	Registered Nurses	Papers were included if they indicated nurses were the study's participants and that these nurses directly cared for dementia patients. This reflects the objective of this review. When studies included other healthcare professionals, nurses must have to be more than 35% of the total study's participants. However, when studies included nurses among other healthcare professionals without mentioning the quantity, the study was excluded. Nursing assistants and nursing students were excluded because they have not received full training and are often still in the process of gaining knowledge, so their knowledge may differ from that of a qualified nurse.
Settings	Acute hospital settings or community‐based practices	Only residential, palliative care and/or primary health settings were included because these settings are involved in caring for dementia patients.
Findings	Clearly indicated/discussed nurses’ knowledge, attitudes or perceptions towards caring for a person with dementia	Articles that sought views from nurses directly so that the analysis could frame what the nurses said rather than reporting what other people think about the nurses.
Designs	All study designs were included	Because this is an exploratory study to identify and analyse what is known about the topic.
Study's quality	High to moderate	Inclusion of low‐quality studies would affect the overall review's findings and conclusion, which may lead to unreliable and inaccurate data.
Date	2010–2021	This allowed for a comprehensive review of development in dementia care over the past decade
Language	English	The study included English papers. It excluded studies in other languages.

### 2.3 Selection of studies

**FIGURE 1:**
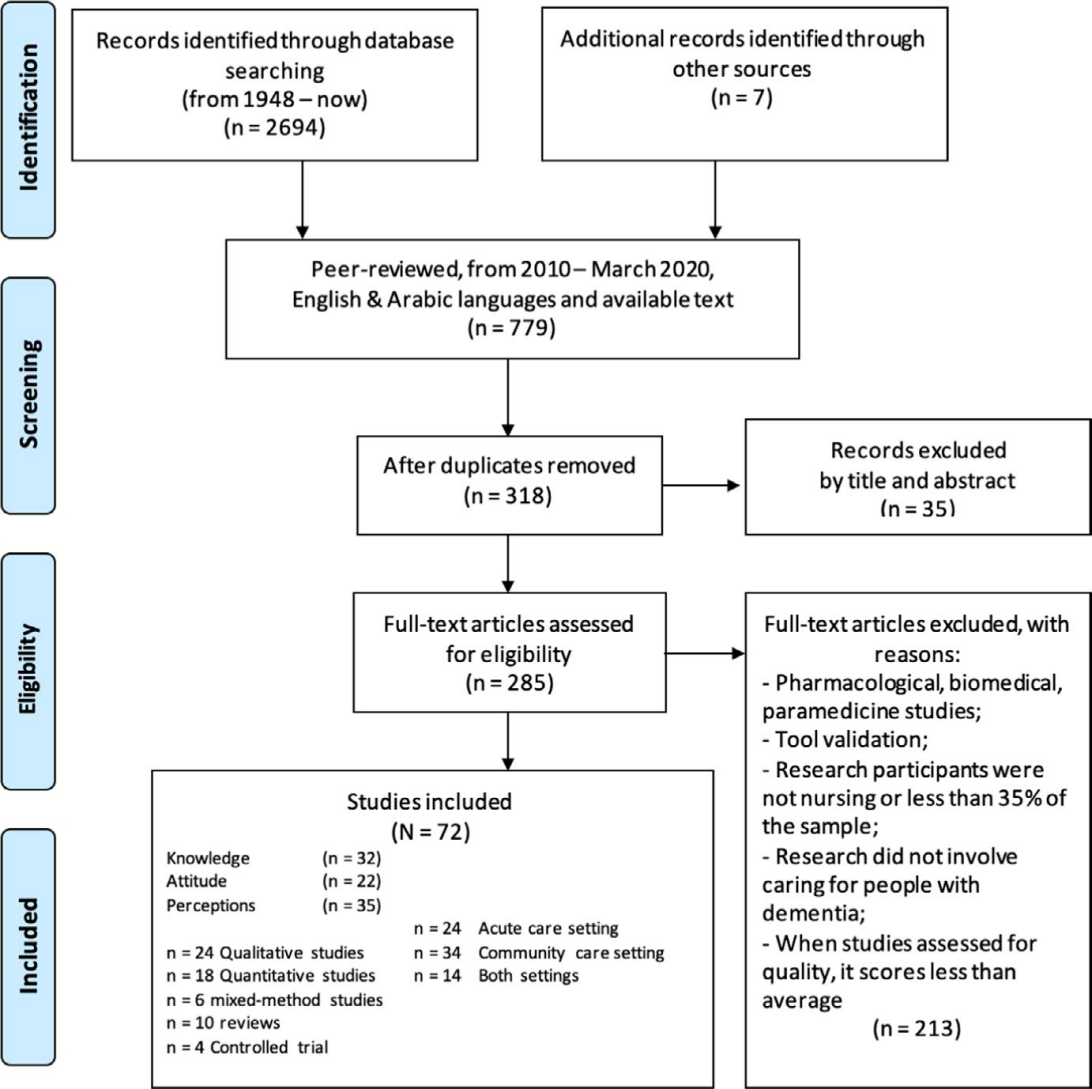
PRISMA chart with search results

The screening of the articles was done by looking at duplicate articles first and then removing them. Following the removal of duplicate articles, the titles of the returned articles were examined. All articles with irrelevant titles were excluded. After the title was reviewed, the abstract of each article was reviewed. This was followed by full‐text screening of the articles by comparing them with the inclusion and exclusion criteria. Two researchers were involved in the screening process, and they finalized the studies after discussion and comparing all the results. The final list of articles was selected after any arguments were resolved through discussion. In case no agreement could be reached, a third expert member of the research team was consulted. The PRISMA flow chart was followed to summarize the screening process (Figure 1).

### 2.4 Methodological quality assessment

It is important to assess the quality of the papers to be included to ensure the integrity of the overall review's findings. The quality of research evidence was assessed using the Joanna Brigs Institute (JBI) appraisal tool for qualitative and quantitative studies, control trials and the mixed‐methods appraisal tool. The tool consists of 10 questions according to different research designs, and the answers need to be chosen from four options: yes, no, unclear and not applicable. This research aims to classify articles according to their quality because high‐quality research papers will be reflective of the validity of the research papers. Studies were considered as having a good quality of research evidence if the score was between 50%–84%. The papers were classified as very high‐quality papers if the score was higher than 85%. In addition, papers scoring 50% or below were rated as low‐quality research evidence. This per cent was set by the study researcher to insure rigorous peer‐reviewed studies were included. The quality appraisal of the articles will be given in the results section.

### 2.5 Data extraction

The data were extracted using a data extraction sheet. The key data extracted from the data include the following: a) author name, title and date of publication, b) setting, c) study design, d) outcome data and e) conclusion. The process of data extraction was completed by the study researcher.

### 2.6 Data analysis

Relevant articles were extracted using NVivo software; the articles were coded accordingly within the software. The extracted data were synthesised and analysed using a thematic analysis method. The main purpose of this form of data synthesis was to assess how the literature addresses the research question. Because the current review focused on the three outcomes of attitude, knowledge and perception, the findings related to these three areas were synthesised by looking at patterns within the dataset. According to authors, the significance of using the thematic analysis method is that it offers a flexible yet rigorous approach to establish links between the study question and test of primary studies (Nicholson et al., 2016). It involves the systematic coding of data and generating of analytical themes. In the present research, line‐to‐line coding was done, followed by grouping together codes based on the similarities and differences between the codes. In this review, the focus was to group together findings into knowledge, attitude and perceptions and to explore their experience when caring for people with dementia across different health regions. As a result of the thematic analysis from the coded studies, three themes were identified: (1) nurses’ knowledge, attitudes and perceptions towards dementia; (2) nursing experience of caring for people with dementia in acute and community care settings; and (3) dementia nursing care across health regions.

## 3 FINDINGS

### 3.1 Included studies

Based on the database search process, a total of 777 articles published between 2010 and March 2021 were obtained. The articles were further screened by looking at duplicate papers. A total of 318 papers were obtained after removing duplicates. After this, the articles were assessed by reviewing the title and abstract. A total of 35 articles were excluded by title and abstract. The remaining 283 articles were reviewed based on inclusion and exclusion criteria. Finally, a total of 72 articles were included in the review. The PRISMA flowchart for the search result is given in Figure 1 above. The list of articles, along with numbering for the selected articles, is provided in Table 3.

The studies were presented in a summary table ordered my World Health Organisation regions and countries, from Europe (Adams et al., 2017; Beck et al., 2017; Blaser & Berset, 2019; Bolmsjö et al., 2012; Brorson et al., 2014; Burns & McIlfatrick, 2015a; Cooper et al., 2016; De Witt et al., 2017; Egede‐Nissen et al., 2017; Ericson‐Lidman et al., 2014; Hansen et al., 2017; Høgsnes et al., 2016; Jakobsen & Sørlie, 2016; Kohler et al., 2016; Krumm et al., 2014; Krupic et al., 2016; Kuehlmeyer et al., 2015; Kupeli et al., 2016; Lee et al., 2017; Lillekroken et al., 2015; McPherson et al., 2016; Midtbust et al., 2018a, 2018b; Naughton et al., 2016; Nilsson et al., 2016; Pellfolk et al., 2010; Pinkert et al., 2018; Rantala et al., 2014; Rosendahl et al., 2016; Ross et al., 2015; Skomakerstuen Ødbehr et al., 2015; Smythe et al., 2014, 2017; Solli et al., 2015; Van Hoof et al., 2016), Americas (Brody et al., 2016; Chaudhury et al., 2017; Daniel et al., 2014; de Witt & Ploeg, 2016; Eritz et al., 2016; Hunter et al., 2017; Karlin et al., 2017; Schindel Martin et al., 2016; Sharpp & Young, 2016; Unroe et al., 2015), the Western Pacific region (Annear, 2020; Conway & Chenery, 2016; Digby et al., 2018; Ervin et al., 2014; Fry et al., 2017; Kable et al., 2015; Lin et al., 2018; McCann et al., 2014; Nakanishi & Miyamoto, 2016; Shannon et al., 2018; Wang, Xiao, et al., 2017; Wang, He, et al., 2017; Wang et al., 2018; Yada et al., 2014), South‐East Asia (Kang et al., 2017) and finally Eastern Mediterranean countries (Bentwich et al., 2017; Yaghmour et al., 2016). Then, the reviews were summarized too (Burns & McIlfatrick, 2015b; Deasey et al., 2014; Digby et al., 2017; Evripidou et al., 2019; Gwernan‐Jones et al., 2020; Machiels et al., 2017; Monthaisong, 2018; Moonga & Likupe, 2016; Tomlinson & Stott, 2015).

### 3.2 Results of the critical appraisal

Using the JBI appraisal tool for quantitative studies (Institute TJB, 2016), 14 studies were found to rank as high quality and six good (refer to Table 3 for details). When the qualitative studies were critically appraised using the JBI checklist (Lockwood et al., 2015), 30 studies were found to be high quality and two were good‐quality papers. Regarding the systematic reviews that were included, the JBI checklist (Aromataris et al., 2015) found six articles were of high quality, while two were good. The mixed‐methods appraisal tool (Pluye et al., 2011) found that all eight studies that used a mixed‐method design were of a high quality. Additionally, control trials were assessed using the JBI critical appraisal tool (Aromataris et al., 2017), which found three good‐quality studies and one high‐quality study. The rationale behind the inclusion of only good‐quality and high‐quality papers is to increase the reliability and validity of these integrated reviews’ findings and the study's rigour.

**TABLE 3:** Summary of included studies

Author & country	Aim	Setting	Sample	Method	Relevant findings	Quality
Beck et al. (2017) UK	Examine nursing home managers’ attitudes, beliefs, knowledge and current practice about advance dementia care planning.	Community care setting	*N* = 116 nursing managers	Quantitative; Cross‐sectional	Nursing homes managers have lack of knowledge towards planning advanced care for dementia patients. Also, they hold negative attitude about the dementia patient's capacity. Nurse–patient with dementia communication is playing important role in changing work environment and care provided to them.	100%
Burns and McIlfatrick (2015a) UK	Assess nurses’ attitudes and knowledge towards pain assessment in dementia patients.	Community care setting *N* = 17	*N* = 33 nurses	Quantitative; Cross‐sectional	Study found that nurses had good knowledge about pain management of dementia patients, but they were uncertain about the safe use of analgesic.	83%
Cooper et al. (2016) UK	Ascertain the consensus in the professional development and care requirements for RNs in UK home care facilities.	Community care setting	*N* = 352 nurses	Quantitative; Cross‐sectional, Delphi survey	Delphi study on nurses working at nursing homes in England, Wales, Scotland & Northern Ireland. Nurses working in nursing homes acquired specific skills, knowledge and experiences. key areas identified for continuous nursing practice development were dementia care, personal care, end‐of‐life care, falls prevention and medication management. Barriers of dementia practice development identified were lack of opportunity for awareness, limited access to training, staff shortage, and lack of management support, besides time and fund burden.	65%
de Witt et al. (2017) UK	Explore hospice, acute care and nursing home nurses’ experiences of pain management for advanced dementia patients in the end‐of‐life settings.	Both settings; *N* = 13 Community *N* = 2 Acute	*N* = 24 nurses	Qualitative; semi‐structured interviews and thematic analysis	Nurses were struggling in administer medication to facilitate pain management to dementia patients. Communication and nurse–physician relationship are important to enhance the care provided to dementia patients. Also, nurses declared that accessing recourses and educational session is important to empower them to learn more and to become more confident in care delivery.	100%
Kupeli et al. (2016) UK	Explore the circumstance approaches and the outcomes for providing good palliative care to people living with advanced dementia in nursing home settings.	Community care setting	*N* = 5 nurses (among *N* = 14 health professionals)	Qualitative; interviews	Prioritization of psychosocial and spiritual care leads to good palliative care. Person‐centred care and end‐of‐life. Poor staff condition, undertrained and negative image of care homes. Negative perception towards work environment with lack of confidence. However, nurses were compassionate towards dementia patients, initiative and find the care is rewarding.	100%
Lee et al. (2017) UK	Explore the perspective of service managers and front‐line care staff on dementia patients.	Both settings	*N* = 19 nurses (among *N* = 54 health professionals)	Qualitative; interviews and focus groups	The staff recognises the important of end of life; however, they mostly face a significant challenge in providing good end‐of‐life care.	80%
McPherson et al. (2016) UK	Explore the experiences of managing. Occupational pressures in front‐line NHS workers attending to geriatric patients living with dementia.	Acute care settings *N* = 3 Dementia	*N* = 10 nurses	Qualitative; interviews	Work pressure effect the nurses’ perception towards dementia patients; it is varied, some show compassionate care and others being burnout and have physiological stress. However, Staff possessing compassion and training may not alter the outcome of care for dementia patients in challenging and demanding work environment.	100%
Naughton et al. (2016) UK	Investigate the development, delivery and early evaluation of the Older Person's Nurse Fellowship programme among senior nurses.	Both settings	*N* = 11 nurses (survey) *N* = 9 nurses (focus group)	Mixed‐method; online postsurvey and focus group interview	Topics such as pharmacology, comprehensive geriatric assessment, frailty and cognitive assessment were regarded as highly relevant and most likely to result in a change to clinical practice. This educational programme focuses on the population rather than the disease. Geriatric nurses contribute in developing a health and social care workforce that is built based on the population needs.	80%
Ross et al. (2015) UK	Investigate nurses’ insight and assistance of patient‐centred care in an acute setting.	Acute care setting Medical	*N* = 14 nurses	Qualitative; semi‐structured interviews	Investigating nursing knowledge & attitude in long‐term facilities in Japan. Dementia knowledge and attitude scores were significantly higher among nurses with experience and higher educational level. Palliative care facilities that had developed manual for end‐of‐life care were scored higher in knowledge and attitude.	100%
Smythe et al. (2014) UK	Assess a brief psychosocial training intervention for healthcare workers working with dementia patients.	Acute care setting	*N* = 81 nurses pre, *N* = 66 post, *N* = 15 interviews	Mixed methods; pre‐ and post‐training questionnaire and focus group	Skills‐based training increases knowledge by improving communication and problem‐solving skills of staff working with dementia patients in acute settings. Acute care settings not ideal for dementia patients. Busy work environment and understaffing were obstacles underpinned by the nurses.	85%
Smythe et al. (2017) UK	Explore the training requirements of nurses working with dementia patients.	Community care setting	*N* = 11 nurses	Qualitative; Focus groups	Barriers to educational; time, training programmes. Valued by residents but not by organisation or at home.	90%
Egede‐Nissen et al. (2017) Norway	Evaluate the minority nurses’ experiences with challenges in caring for dementia patients.	Community care setting	*N* = 5 nurses	Qualitative; interviews, narrative approach	In a phenomenological–hermeneutical study, five nurses’, from different cultural background, perceptions were structurally analysed. When dementia patients develop communication difficulties, nurse–patient relationship is affected and the challenge arises. these challenges comprise both ethical and cultural striving to understand persons with dementia to care for persons with dementia in an unfamiliar context may be understood as a striving for acting ethically, when at the same time striving to adapt and acculturate to new cultural norms, in order to practice good dementia care.	80%
Hansen et al. (2017) Norway	Explore the psychosocial needs of persons with dementia.	Community care setting	*N* = 24 nurses	Qualitative; semi‐structured focus group interviews	Study highlighted the importance of including the psychological and spiritual care alongside with the physical care. providing holistic care for dementia patients is enabling them to live at their own place as long as possible. This is by raising the awareness of nurses to meet their psychological need.	80%
Jakobsen and Sørlie (2016) Norway	Explore the caregiver's experiences with ethical challenges in dementia care settings and the importance of professional leadership.	Community care setting	*N* = 19 nurses (among *N* = 23 health professionals)	Qualitative; narrative interviews. a phenomenological–hermeneutical	The concept of trust and mistrust were discussed. As leadership influencing the attitude towards caring for dementia patients. Most nurses act negatively towards their work as a result of the negative work atmosphere and leaders’ perspectives towards their staff.	70%
Lillekroken et al. (2015) Norway	Explore nurses’ approaches that may support the sense of coherence in dementia patients.	Community care setting *N* = 2	*N* = 16 nurses	Qualitative; focus group interviews	The sense of coherence can be developed. More awareness needed. Caring, finding creative solutions and meaningful activities enhance the person's with dementia sense of coherence.	90%
Midtbust et al. (2018b) Norway	Explore the barriers of healthcare professionals when caring for people with dementia in palliative care settings.	Community care settings *N* = 4 nursing homes	Four focus groups and *N* = 20 in‐depth interviews	Qualitative; focused group and interviews	Lack of communication was experienced as the main barrier to facilitating dementia care. Work and pressure impact negatively on caring for people with dementia, especially on the weakest and bedridden patients with dementia. Conflict feelings were found among nurses as a result of wanting to spend more time with every patient to provide their care and the burnout and feeling pressure to help everyone. Priority was always given to dying residents. Organisational changes are suggested in terms of staffing to minimise nurse's burnout.	80%
Midtbust et al. (2018a). Norway	Explore healthcare professionals experience when caring for patients with severe dementia.	Community care settings	*N* = 20 Nurses	Qualitative; interviews	Nurses’ with limited knowledge about dementia increase their frustration and caring difficulties. Nurses are unable to provide care for patients with severe dementia as result of their *suffering*. Most challenges were attributed to the behavioural symptoms of dementia, like agitation, irritability, physical aggression and rejection of care. Nurses described their experience as painful and distressing as they found themselves helpless. Getting to know patients more is one of the successful approaches in providing care and minimising patient irritations. The important of having close relative around to aid the care process.	80%
Skomakerstuen Ødbehr et al. (2015) Norway	Analyse nurses’ attitudes in regard to accommodations of patients’ religious inclinations and practice in dementia care.	Community care setting *N* = 4	*N* = 16 nurses	Qualitative; Eight focus group interviews	Embarrassment versus comfort towards resident's religiosity private matters. Difficult discussing death and End‐of‐life issues. focus on life and quality of life.	90%
Solli et al. (2015) Norway	Investigate the relationship between nurses and caregivers using a web camera and web forum as a communication strategy.	Community care setting Web	*N* = 6 nurses	Qualitative; content analysis	Nurses perception towards dementia patients affects the person‐centred care. Perceive the dementia patients as demanded.	100%
Bolmsjö et al. (2012) Sweden	Investigate the application of drama as a tool to support rejection among workers in the nursing homes for dementia patients.	Community care setting	*N* = 10 nurses	Qualitative; observations, focus group and reflexive diary	Drama and theatrical training are useful in enhancing knowledge among nurses caring for dementia patients. Reflection on daily care practice is influenced by exercising.	70%
Brorson et al. (2014) Sweden	Explore nurses’ experiences on end‐of‐life pain relief in dementia patients.	Community care setting	*N* = 7 nurses	Qualitative; Semi‐structured interview technique with open‐ended questions	The paper explored the end‐of‐life care for dementia patients in a palliative care centre and how is the pain management. Pain management perceived as difficult task for nurses providing care for a dementia patient. As nurses feel powerless and unable to communicate with them. Nurses concern about the recourses available such standardised pain management tools and medication.	90%
Ericson‐Lidman et al. (2014) Sweden	Investigate care providers' lived experiences in caring for dementia patients.	Community care setting	*N* = 12 nurses	Qualitative; semi‐structured interviews	Nurses struggled to perform person‐centred care for dementia patients as most did not hold positive attitudes towards dementia patients. work environment impact on nurses’ attitude towards dementia patients.	90%
Høgsnes et al. (2016) Sweden	Investigate the perception of end‐of‐life care by healthcare professionals in records of deceased dementia patients in Swedish nursing homes.	Both settings Databases	*N* = 50 nursing records	Qualitative; retrospective approach	Investigate end‐of‐life in nurses’ documentation. The end‐of‐life care has been depicted in the healthcare records based on factors such as the participation and communication and decision‐making, assessment and prevention of symptom and following up after the resident has died. Physical symptoms have been recorded and, to a lesser extent, psychological or existential/spiritual needs. Healthcare professionals must have a holistic approach to the dementia patient.	100%
Krupic et al. (2016) Sweden	Explore nurses’ perspectives about pain management for people with dementia in postoperative setting.	Acute care setting (operation)	*N* = 51 nurses	Qualitative content analysis of self‐administered questionnaire	Nurses mostly rely on the facial expression and the body language of the person with dementia to recognise their pain, while they agreed that with the severity of dementia this becomes more challenging. Communication with the person with dementia difficult. Nurses acknowledge the lack of awareness in providing care for people with dementia.	90%
Nilsson et al. (2016) Sweden	Illuminate the meanings of caring for geriatric patients with cognitive impairment in acute care settings.	Acute care setting Medical, Oncology & Neurology	*N* = 31 nurses	Qualitative; interviews. phenomenological‐hermeneutic	Caring for dementia patient is complicated. Gap between thoughts and perceptions.	100%
Pellfolk et al. (2010) Sweden	Effect on an educational programme on nurses’ knowledge, attitudes and practice.	Community care settings *N* = 40 dementia wards	Pre‐intervention (*N* = 205, *N* = 188 staff) Postintervention (*N* = 156, *N* = 133)	Cluster randomised controlled trial	Nurses knowledge in intervention group was higher than the control group in follow‐up but not in no different in their attitudes. Overall, knowledge and attitudes scores were significantly improved by the educational programme. Nurses minimises the use of restrains after completing the education programme.	50%
Rosendahl et al. (2016) Sweden	Evaluate the experiences of family caregivers and professionals on the care provided to immigrants with dementia.	Community care setting	*N* = 9 nurses	Qualitative study; semi‐structured interviews	Family members are playing a crucial role in facilitating communication of immigrants’ dementia patients between the nursing staff and the dementia patients. Family also enable accessing the cultural activities that the dementia patients want, which professional caregivers were either not able to recognise as needed or could not deliver.	80%
Adams et al. (2017) The Netherlands	Explore perceptions of the needed expertise and assess the variations in job satisfaction and motivation.	Community care setting	*N* = 138 nurses	Quantitative; Cross‐sectional	Job satisfaction and motivation were different in nurses caring for dementia patients in different nursing homes types. Most significantly, correlate with higher job satisfaction and motivation is social support.	100%
Van Hoof et al. (2016) The Netherlands	Examine the factors determining the sense of home setting for geriatric patients in the nursing home from the perspective of professional caregivers including nurses.	Community care setting *N* = 4	*N* = 26 nurses	Qualitative; photography, interviews and focus groups	Communication. Empathy and aggression behaviour. Nurses exclude dementia patients from decision‐making.	100%
Krumm et al. (2014) Germany	Expound health professionals’ experiences of assessing the symptoms dementia.	Community care setting *N* = 3	*N* = 13 nurses	Qualitative; semi‐structured interviews	Implementation of the Minimal Documentation system for Palliative care; the tool enhances the quality of care of dementia patients.	90%
Kuehlmeyer et al. (2015) Germany	Determine nurses’ perceptions and assess the non‐verbal behaviour about feeding dementia patients.	Community care setting *N* = 12	*N* = 131 nurses	Quantitative; Cross‐sectional	Nurses consider the non‐verbal communication of the dementia patients as crucial in decision‐making process.	71%
Pinkert et al. (2018) Germany and Austria	Explain the experiences of nurses’ caring for people with dementia.	Acute care settings Mixed	Germany: *N* = 22 nurses among *N* = 42 healthcare professionals. Austria: *N* = 57 nurses.	Quantitative; Focus group	Training correlates with knowledge and meeting patients’ needs. Nurses found that caring for people with dementia is rewarding. Nurses in both countries were greatly uncertain about the care and perceived a person with dementia. Having person with dementia disturbs the ward's routine. In order to perform person‐centred care, organisations must minimise restrains on nurses. Some nurses believed that they are creative in providing care for the person with dementia that makes their care rewarding. Application of dementia‐friendly environment proves its benefit in minimising patient's confusion.	100%
Rantala et al. (2014) Finland	Explore perceptions of nurses on the barriers to postoperative pain management in hip fracture dementia patients.	Acute care settings *N* = 7	*N* = 331 nurses	Quantitative; Cross‐sectional	Difficulty in assessing pain for dementia patients. There were statistically significant differences between the sufficiency of pain management and barriers.	83%
Blaser and Berset (2019) Switzerland	Investigate nurses’ attitudes when caring for people with dementia.	Both care settings	*N* = 417 nurses	Quantitative; cross‐sectional	Nurses attitudes were significantly positive with nurses working in dementia‐related wards. The care setting (ward) has strong association with attitudes scores. All other factors– such as age, gender, years of experience, nursing degree and employment– were not found to be associated with the attitudes scores (no effect found).	86%
Kohler et al. (2016) Switzerland	Explore the effectiveness of educational interventions on urinary incontinence and quality of life for dementia patients.	Community care setting *N* = 7	*N* = 140 nurses	Randomized control trial; stepped‐wedge trial design	an educational programme and case conferences during 6 months proved to be elective in reducing urinary incontinence and improve the quality of life.	69%
Brody et al. (2016) USA	Test the ability of the DSM‐H programme to ameliorate the confidence, attitude and knowledge of nurses among other healthcare professionals in cognitive impairment pain management.	Community care setting	*N* = 143 nurses (among *N* = 191 health professionals)	Quantitative; pre‐ and postquestionnaire	Evaluating educational programme (DSM‐H). Significant improvement in pain knowledge and confidence, depression knowledge and confidence d neuropsychiatric symptom. Attitudes, Intervention knowledge and confidence.	88%
Daniel et al. (2014) USA	Expound on nurses’ practice and expertise level and comfort in the management of dementia patients.	Both settings	*N* = 114 nurses	Quantitative; Cross‐sectional	Wide diversity in practice and skill among nurses about dementia screening, evaluation and treatment. slightly over half of the nurses in this sample who care for adult patients were comfortable and familiar with the techniques for screening for dementia, diagnosing dementia, or managing patients with dementia.	57%
Karlin et al. (2017) USA	assess the evolution of a training programme, process and document its outcome.	Both settings	*N* = 32 nurses and care managers	Quantitative; pre‐ and postintervention questionnaire	a robust transformation in knowledge, attitudes and self‐efficacy after training participants, with the greatest impact on knowledge and self‐efficacy to manage behaviours.	71%
Sharpp and Young (2016) USA	Describe the healthcare occurrences and experiences of residents and caregivers transferred from assisted living to the emergency settings.	Acute care settings	*N* = 14 nurses	Mixed‐method study; quantitative demographic information, interviews and focus group	Quantitative data and Focus group with an unspecified number of participants. Geriatric person claimed to be neglected by nurses in the acute hospital settings. Fall was the major problem faced the nurses that caused agitation and frustration for them.	80%
Unroe et al. (2015) USA	Make comparison between palliative care knowledge and practices of staff.	Community care setting *N* = 51	*N* = 1163 nurses	Quantitative; Cross‐sectional	Investigating the relationship between nursing homes and nurses’ profile in a cross‐sectional survey study using The Palliative Care Survey validated tool. Nursing assistants were significantly lower in knowledge and practice than practical nurses and Registered Nurses. Among all study participants, the physical symptoms and changes in knowledge were high. The end‐of‐life knowledge was remark low in all study participants. However, nurses working in hospice scored higher.	100%
Chaudhury et al. (2017) Canada	Analyse the effectiveness of settings alterations on residents’ mealtime experience and staff practice in care units.	Community care setting *N* = 2	*N* = 17 nurses	Qualitative; pre‐ and postrenovation ethnographic observations	The physical environment plays a crucial role in enhancing dining experience for dementia patients. Person‐centred care enhances the care.	90%
de Witt and Ploeg (2016) Canada	Expound on healthcare practitioners’ experiences in caring for the geriatric dementia patients.	Both settings	*N* = 9 nurses (among *N* = 15 health professionals)	Qualitative; Interviews	The endings strongly supported providing healthcare educational programmes and continuing professional development initiatives in the principles of person‐centred approaches. Most participants declared that they are providing the best care as they can. Emotional struggles, cultural changes and holistic approaches were suggested by participants in community care settings.	90%
Eritz et al. (2016) Canada	Analyse nurses’ empathy, perceived patient‐centred approach and aggressive behaviours.	Community care setting *N* = 6	*N* = 99 nurses	Mixed method; controlled, parallel randomized groups design	Negative correlation between nurses’ perceptions towards personhood of residents and person's cognitive impairments. Communication and personal interactions positively changed after the intervention.	80%
Hunter et al. (2017) Canada	Get insight on the safety in rural Emergency settings for transitional care for community‐dwelling geriatric with dementia from the view of healthcare practitioners.	Acute care settings *N* = 2	*N* = 12 health professionals including nurses	Qualitative; interviews and field notes	Safety and environment. Knowledge and process (practice). Pressure, cannot control and burnout (work).	100%
Schindel Martin et al. (2016) Canada	Assess the influence of Gentle Persuasive Approaches (GPA) educational in the improvement of dementia care intervention.	Acute care settings medical, surgical, oncology, orthopaedic, intensive, cardiology & emergency	*N* = 468 nurses	mixed‐methods; non‐randomized controlled; a quasi‐experimental design with repeated measures and focus groups	Skills and knowledge. Experience.	85%
Annear (2020) Australia	Explore the healthcare professional's knowledge about dementia.	Both settings	*N* = 99 Nurses (among *N* = 234 healthcare professionals)	Quantitative; cross‐sectional	Knowledge deficit observed for the risks and health promotion subscale and items addressing blood pressure, influence of lifestyle factors, prevalence of vascular dementia and differentiation of cognitive symptoms. Participants shows lack of understanding of the dementia risk factors. Participants who undertook educational programme about dementia were more knowledgeable about it and the care process. Weak association between years of experience and knowledge level.	100%
Conway and Chenery (2016) Australia	Evaluate the effectiveness of a communication skills training programme on community nurses’ knowledge.	Community care setting *N* = 12	*N* = 38 nurses	Quantitative; Controlled pre‐ and post‐test	Application of communication skills training in community‐based aged care practice can contribute to quality dementia care.	62%
Digby et al. (2018) Australia	Explore nurses’ perspectives when caring for people with dementia in order to critically investigate the poor care received that reported widely by patients.	Acute care settings Geriatric rehabilitation	*N* = 29 Nurses	Qualitative; interviews	Nurses showed that they prioritized patients with rehabilitation goals who were returning to the community ahead of others who may be awaiting a bed in residential care. Patients with comorbidities were the least concerned by nurses. It was clear from the interviews that not all nurses perceived patients equally. Most nurses perceived the person with dementia as *unworthy* (lack of value, undeserving, not good enough, inappropriate) to the ward environment. Nurses professed that people with dementia as disturbance of the work routine and environment. Work organisation adding pressure to nurses.	80%
Ervin et al. (2014) Australia	Investigate nurses’ perceptions of non‐pharmacological and pharmacological approaches in dementia management.	Community care setting *N* = 6	*N* = 130 nurses	Qualitative; questionnaire	Non‐pharmacological interventions are not nurses’ role. Nurses are always under pressure and lack of time cause their burnout.	100%
Fry et al. (2017) Australia	Investigate emergency nurses’ perceptions of the Pain Assessment in Advanced Dementia in geriatric with cognitive impairment.	Acute care setting *N* = 3 Emergency	*N* = 36 nurses	Qualitative; Focus group interviews	Challenge in identifying, managing and evaluating pain. Use of PAINAD was recommended by nurses than other similar tools available that ease the challenges.	100%
Kable et al. (2015) Australia	To expound health professionals’ position on discharge planning and transitional care for dementia patients.	Acute care setting Medical	*N* = 16 nurses (among *N* = 33 health professionals)	Qualitative; focus groups	Study claimed that nurses’ perceptions greatly affect systems failures in transitional care for dementia patients. Nurses perceptions greatly affected among other issues, systems failures in transitional care for patients with dementia.	100%
Shannon et al. (2018) Australia	Explore nursing care for people with dementia.	Acute care setting Rural hospital	*N* = 19 Nurses (Observations *N* = 13 Nurses and interviews *N* = 19 Nurses)	Qualitative; observations and interviews	Nurses trying their best to make consistent ward routine to maintain calm atmosphere and allow focusing on maintaining patient's dignity. Nurses used physical and chemical restrains on patients with dementia to keep them safe and avoid their falling. Person‐centred care is challenging. Nurses referred to the importance of having a family member to support the care and some seeks help from security personnel. Nurses main concern is the patient safety and to minimise risks.	80%
McCann et al. (2014) Australia	Examine the attitudes of clinical staff towards the management of aggression in acute geriatric psychiatry inpatient environments.	Acute care settings *N* = 3 Psychiatry	*N* = 75 nurses	Quantitative; Cross‐sectional	Aggressive behaviours of dementia patients cause difficulty in developing bonds and communication. Using medication useful for managing aggressive behaviours.	100%
Nakanishi and Miyamoto (2016) Japan	Focus on the factors associated to attitudes and knowledge of nursing facility staff linked to palliative care of advanced dementia care.	Community care setting *N* = 74	*N* = 121 nurses (among *N* = 275 health professionals)	Quantitative; Cross‐sectional	A basic knowledge about dementia was noted. Dementia knowledge and attitudes scores were found to be significantly higher among nurses that had more experience and possessed higher educational levels. Community care settings with nurses that have developed manual end‐of‐life care skills scored higher in terms of knowledge and attitudes.	66%
Yada et al. (2014) Japan	Provide insight into the precise work‐related stress in psychiatric dementia nurses care for geriatric patients demonstrating behavioural and psychological symptoms.	Acute care settings *N* = 2 Psychiatry	*N* = 244 nurses	Quantitative; Cross‐sectional	If the workplace environment stressors were reduced for nurses, the cognitive health of the dementia patients is improved. Nurses working with dementia patients have high stress level and burnout. Shouting of the dementia patients is considered one of the environmental stressors that claimed by nurses. Shouting is common in such ward, in addition to the odour and noise.	100%
Wang, He, et al. (2017) China	Ascertain the effectiveness of a nurse‐led dementia educational and knowledge and perception.	Community care setting *N* = 14	*N* = 68 nurses (among *N* = 170 health professionals)	Cluster randomised controlled trial	Nurse‐led dementia educational and knowledge translation programme positively improve attitude and knowledge. Educational background impacts the dementia care practices.	77%
Wang, Xiao, et al. (2017) China	Ascertain the effectiveness of dementia‐septic educational programme incorporating WeChat‐based learning interactions could improve nurses’ dementia attitudes, knowledge and intentions.	Community care setting	*N* = 115 nurses	Randomized controlled trial	demonstrated an educational programme using WeChat application. Knowledge an attitude was positively improved. The programme shows its acceptability and practicability in improving nurses' dementia attitudes and knowledge and intentions to achieve early detection of dementia.	69%
Wang et al. (2018) China	Assess healthcare professional knowledge and attitudes towards people with dementia in community care settings.	Community care settings	*N* = 178 Nurses (*N* = 390 healthcare professionals)	Quantitative; cross‐sectional	Generally, positive attitudes towards dementia. Low dementia knowledge noted; especially in questions related to symptoms and caregiving. Failed to apply person‐centred care.	75%
Lin et al. (2018) Taiwan	Examine dementia care knowledge, attitude and behaviour among nurses about dementia care and self‐education.	Acute care settings Emergency, psychiatry, and neurology	*N* = 387 Nurses	Quantitative; cross‐sectional	Knowledge scores were significantly associated with age, nursing experience, accreditation as Registered Nurse, holding a bachelor's degree, work unit, training courses and learning behaviour towards dementia care. Emergency nurses reported a significant low knowledge about dementia care that is significantly lower than the psychiatric and neurology ward nurses. Emergency nurses were also significantly lower percentage undertook dementia care training and actively searched for information on dementia care.	75%
Kang et al. (2017) South Korea	Investigate the impact of an educational programme on acute care nurses in the aspect of dementia knowledge, their attitude and the role of caregivers.	Acute care settings Medical	*N* = 40 nurses’ educational programme *N* = 12 nurses’ interviews	mixed‐methods sequential explanatory design; single group, pre‐ and postdesign and individual interviews	Educational alter self‐confidence lead to improve assessment for dementia patients. Improve knowledge and enhance positive attitude.	75%
Bentwich et al. (2017) Palestine	Explore the existing gaps between formal dementia careers and their perspective of dementia people dignity and autonomy.	Both settings *N* = 3 Community *N* = 1 Acute	*N* = 196 nurses	Quantitative; Cross‐sectional	Significant variations in the attitudes to autonomy and human dignity patients living with dementia. Nurses lacked positive perception and so did not maintain the dignity and autonomy of dementia patients in their care.	50%
Yaghmour et al. (2016) Saudi Arabia	Provide insight into the knowledge of nurses on dementia.	Acute care settings *N* = 2 Psychiatry & acute	*N* = 265 nurses	Quantitative; Cross‐sectional	Poor understanding of dementia in Saudi Arabia is linked to nurses' unresponsiveness to geriatric patient with cognitive impairment such as depression and delirium. Nurses rated their knowledge low which significantly correlates with their knowledge score.	85%
Burns and McIlfatrick (2015b) UK (Canada, Australia and US)	Explore the evidence on nurses’ attitudes and knowledge in pain assessment in dementia geriatric patients.	Both settings	*N* = 11 studies (7 to 244 nurses)	Systematic narrative review between 2000–2014	Nurses faced challenge in diagnosing pain for dementia patients. There are inadequate pain assessment tools available for nurses to use. Nurse–physician relationship is important to assist in communicating and investigating pain for dementia patients.	73%
Deasey et al. (2014) Australia	Explore nurses' attitude, knowledge of the ageing processes in geriatric patients in the emergency care environment.	Acute care setting Emergency	*N* = 16 studies	Literature review Between 2004–2012	Lack of nurses’ knowledge effect the care provided to the dementia patients. The dementia patients become vulnerable as a result of lack of understanding of the disease process and skills. Negative attitude of nurses towards dementia patients associated with his level of dependency.	91%
Digby et al. (2017) Australia (UK, Sweden, Australia, Ireland, US, Japan, Finland and Malta)	Explore of literature about the perception of nurses and people living with dementia on acute hospital care.	Acute care setting	*N* = 24 qualitative studies; (*N* = 270 nurses)	Integrative review between 2005–2015	Dementia patients treated in the acute hospital setting consider being a disturbance to the ward routine and mostly disregarded. Dementia patients are given low priority and have been regarded as an interruption to normal routine at the hospital. There is an absence of a patient‐centred approach in caring for dementia patient. Inadequate nursing support leads to low job satisfaction of nurses caring for dementia patients.	90%
Evripidou et al. (2019) Cyprus (UK, Norway, Sweden, the United States, Australia, Korea and Palestine)	Determine nurses understanding, knowledge and attitude towards dementia patients.	Both settings	*N* = 19 studies	Systematic literature review	Nurses lack in their knowledge about dementia with negative attitude towards dementia patients that impact negatively on the care provided.	82%
Gwernan‐Jones et al. (2020) UK	Review and synthesise qualitative data from studies exploring the experiences of hospital staff who care for people living with dementia.	Acute care settings	*N* = 58 qualitative studies	Qualitative systematic review	Person‐centred care improves experiences of care for people with dementia and their carers; also improve hospital staff experiences when caring for them. Person‐centred care can reduce moral distress related to caring for people with dementia and improve job satisfaction for hospital staff. An important aspect of training involves attributing responsive behaviours to unmet needs. Time spent get‐ ting to know individual people with dementia is valuable because it can prevent or resolve responsive behaviour. Providing staff training may be inadequate to effectively enable person‐centred care; hospital cultures that prioritize psychological well‐being of people with dementia at the same level as physical health are needed to enable staff to spend time getting to know people with dementia.	100%
Machiels et al. (2017) The Netherland	Provide a current update on communication interventions approaches in daily nursing care activities, in the home care setting and their outcomes in dementia care by nurse professionals.	Community care setting	*N* = 6 RCTs studies; (*N* = 235 nurses)	Systematic literature review Between 2000–2016	All studies that measured non‐verbal and verbal communication, found positive effects on at least some of the communication outcomes. Communication is important factor to establish patient–nurse relationship. Most people with dementia admitted in the long‐term facilities have difficulties in communication.	82%
Monthaisong (2018) USA (6 UK, 3 Sweden, 2 Norway, 2 Ireland, 2 Australia, Finland, Malta, France, Belgium)	Investigate nurses’ experiences towards caring for dementia patients.	Both settings	*N* = 19 studies	Integrated literature review	looked at nurses’ experiences and perception towards dementia patients. Nurses combating painful emotions when caring for dementia patients. Many nurses complain about working environment and the work pressure. Nurses recognise the need of meeting the holistic care for dementia patients. They are experiencing inadequate knowledge and competence of dementia. These consider as barriers of caring for a dementia patient in any setting.	60%
Moonga and Likupe (2016) UK (8 UK, 4 Australia, 3 Sweden, 1 Ireland)	Probe the experiences of healthcare practitioners providing care to dementia patients in orthopaedic ward.	Acute care setting‐ Orthopaedic	*N* = 14 studies	Systematic review Between 1998–2013	Educationally intervention improves nursing practice and coping mechanism. Person‐centred approaches minimise nurses’ burnout. Educational and practice together improve.	80%
Saxell and Ingvert (2019) Sweden	Describe nurses experiences of facilitators for the delivery of person‐centred care to inpatients with dementia.	Acute care settings	*N* = 19 studies	Systematic review	Internal facilitators (experience and knowledge; values and beliefs; professional identity; empathy) External facilitators (physical environment; organisational culture and structure). Facilitating actions (forming a holistic picture; establishing trust; adjusting routines and interventions). While facilitators did exist in the hospital setting, the findings indicate that care received by inpatients with dementia is dependent on individual registered nurses knowledge, personal attitude and ability to compensate for structural flaws.	90%
Tomlinson and Stott (2015) UK	Investigate attitudes and factors involved in assisted dying of dementia.	Both settings	*N* = 18 studies	Systematic literature review Between 1992–2013	Restricted views towards end‐of‐life. Opinion varies according to the severity of dementia, religion, ethnicity and gender.	90%

### 3.3 Characteristics of the studies

The characteristics of selected studies and its context, such as scope, setting and participants’ numbers are presented in Table 3.

### 3.4 Review's findings

#### 3.4.1 Theme 1: Nurses’ knowledge, attitudes and perceptions towards dementia

The review identified 72 studies that had investigated and explored nurses’ *knowledge* (Annear, 2020; Bentwich et al., 2017; Bolmsjö et al., 2012; Brody et al., 2016; Burns & McIlfatrick, 2015a, 2015b; Conway & Chenery, 2016; Cooper et al., 2016; Daniel et al., 2014; de Witt & Ploeg, 2016; Deasey et al., 2014; Digby et al., 2017; Evripidou et al., 2019; Kang et al., 2017; Karlin et al., 2017; Kohler et al., 2016; Lin et al., 2018; Monthaisong, 2018; Moonga & Likupe, 2016; Nakanishi & Miyamoto, 2016; Naughton et al., 2016; Pellfolk et al., 2010; Pinkert et al., 2018; Saxell et al., 2019; Schindel Martin et al., 2016; Smythe et al., 2014, 2017; Unroe et al., 2015; Wang, Xiao, et al., 2017; Wang, He, et al., 2017; Wang et al., 2018; Yaghmour et al., 2016), *attitudes* (Bentwich et al., 2017; Blaser & Berset, 2019; Brody et al., 2016; Burns & McIlfatrick, 2015a; Deasey et al., 2014; Digby et al., 2017, 2018; Ericson‐Lidman et al., 2014; Eritz et al., 2016; Evripidou et al., 2019; Gwernan‐Jones et al., 2020; Høgsnes et al., 2016; Jakobsen & Sørlie, 2016; Kang et al., 2017; Kuehlmeyer et al., 2015; Machiels et al., 2017; McCann et al., 2014; Monthaisong, 2018; Nakanishi & Miyamoto, 2016; Nilsson et al., 2016; Pellfolk et al., 2010; Saxell et al., 2019; Shannon et al., 2018; Sharpp & Young, 2016; Tomlinson & Stott, 2015; Wang, Xiao, et al., 2017; Wang et al., 2018) and *perceptions* (Adams et al., 2017; Bentwich et al., 2017; Brody et al., 2016; Brorson et al., 2014; Chaudhury et al., 2017; De Witt et al., 2017; de Witt & Ploeg, 2016; Digby et al., 2018; Egede‐Nissen et al., 2017; Eritz et al., 2016; Ervin et al., 2014; Fry et al., 2017; Hansen et al., 2017; Kable et al., 2015; Kim & Lee, 2017; Krumm et al., 2014; Krupic et al., 2016; Kupeli et al., 2016; Lillekroken et al., 2015; McPherson et al., 2016; Midtbust et al., 2018a, 2018b; Monthaisong, 2018; Moonga & Likupe, 2016; Nilsson et al., 2016; Pinkert et al., 2018; Rantala et al., 2014; Rosendahl et al., 2016; Ross et al., 2015; Shannon et al., 2018; Skomakerstuen Ødbehr et al., 2015; Solli et al., 2015; Van Hoof et al., 2016; Yada et al., 2014).

Several studies reported that most nurses possessed the basic knowledge of dementia (Beck et al., 2017; Bentwich et al., 2017; Burns & McIlfatrick, 2015a; Cooper et al., 2016; Daniel et al., 2014; Deasey et al., 2014; Ericson‐Lidman et al., 2014; Evripidou et al., 2019; Nakanishi & Miyamoto, 2016; Naughton et al., 2016; Unroe et al., 2015; Yaghmour et al., 2016), with a good understanding of effective screening and diagnosing of dementia and sufficient knowledge of general pain management (Burns & McIlfatrick, 2015a; Daniel et al., 2014; Naughton et al., 2016; Unroe et al., 2015). However, there were also studies that highlighted significant deficiencies in nurses’ knowledge about dementia; this was frequently highlighted within the literature (Beck et al., 2017; Bentwich et al., 2017; Deasey et al., 2014; Ericson‐Lidman et al., 2014; Evripidou et al., 2019; Yaghmour et al., 2016). Accurate knowledge of the disease spectrum from onset to end of life was found to be remarkably low (Brorson et al., 2014; Unroe et al., 2015; Wang et al., 2018). There was also a lack of knowledge about the specific safe use of certain pain management therapies (Burns & McIlfatrick, 2015a; Rantala et al., 2014) and a lack of understanding of the disease process and skill set needed for the disease's management (Annear, 2020; Burns & McIlfatrick, 2015a; Deasey et al., 2014; Lin et al., 2018; Naughton et al., 2016; Unroe et al., 2015).

The significance of these studies is that they revealed a positive relationship between knowledge and dementia care. For example, nurses with adequate knowledge of dementia were generally found to have a more positive attitude towards dementia and dementia care; however, this did not always translate into competency in the teams providing quality care for dementia patients (Deasey et al., 2014; Nakanishi & Miyamoto, 2016). Also, the negative attitudes of nurses towards dementia patients were found to be associated with high levels of perceived patient dependency (Deasey et al., 2014; Evripidou et al., 2019) with some nurses in acute care settings reporting feeling hesitant to attend to patient cases related to old age (Deasey et al., 2014; Digby et al., 2017). Dementia knowledge and attitudes scores were found to be significantly higher among nurses who had more experience and possessed higher educational levels (Blaser & Berset, 2019; Nakanishi & Miyamoto, 2016). This is consistent with the findings from Norway, which stated that licensed nurses with higher work experience had higher dementia care knowledge scores (Jakobsen & Sørlie, 2016).

Studies also reported about an improvement in knowledge and attitude after the provision of training. To this end, nurses’ knowledge and perceptions were significantly improved when educational interventions (Brody et al., 2016; de Witt & Ploeg, 2016; Kang et al., 2017; Kohler et al., 2016; Pellfolk et al., 2010; Wang, Xiao, et al., 2017; Wang, He, et al., 2017) and training programmes (Bolmsjö et al., 2012; Conway & Chenery, 2016; De Witt et al., 2017; Eritz et al., 2016; Fry et al., 2017; Karlin et al., 2017; Krumm et al., 2014; Smythe et al., 2014) were implemented in both community and acute care settings in a number of different regions, including the Americas (Canada and United States), Europe (Germany, Sweden, Switzerland, UK, Austria, Finland, Italy and Norway), Western Pacific (Australia, China and Taiwan) and South‐East Asia (South Korea).

Hence, informed by these studies, researchers have recommended on‐the‐job training to increase the knowledge and attitudes of nurses. The literature review revealed positive benefits of educational programmes in improving knowledge. In the United States, an educational programme for community nurses provided significant improvements in levels of pain knowledge, neuropsychiatric symptom recognition and depression knowledge (Brody et al., 2016). In Canada, the Gentle Persuasive Approaches educational programme was carried out to educate nurses in acute care settings, including medical, surgical, oncology, orthopaedic, intensive, emergency wards and cardiology care units; this programme was found to significantly enhance nurses’ knowledge, perceptions and practices about dementia (Schindel Martin et al., 2016). Similarly, studies done in Australia, South Korea and China also revealed an improvement in attitude and knowledge towards dementia patients (Annear, 2020; Kang et al., 2017; Wang, Xiao, et al., 2017; Wang, He, et al., 2017). Therefore, these approaches can be useful in maximizing the advancement of interprofessional collaboration, thereby improving the overall dementia care for the patient.

#### 3.4.2 Theme 2: Nursing experience of the factors influencing dementia care in acute and community care settings

Work environment and care settings were believed to play a crucial role in the nurses’ perceptions and in their dementia care delivery. A wide diversity in practices along with the skills of the nurses working in both community and acute care settings was evident with respect to dementia screening, evaluation and treatment (Daniel et al., 2014; Van Hoof et al., 2016). In community care settings, researchers have suggested the application of effective communication skills at the time of training community‐based caregivers, thereby contributing to the quality care of dementia‐afflicted individuals (Conway & Chenery, 2016; Eritz et al., 2016; Gwernan‐Jones et al., 2020; Smythe et al., 2014, 2017). Additionally, community care settings with nurses who have developed manual end‐of‐life care skills scored higher in terms of knowledge and attitudes (Nakanishi & Miyamoto, 2016). Contextually, at the time of assessing the barriers with respect to the development of dementia care, it was found that the lack of educational opportunity, limited access to training, staff shortages, lack of management support, time constraints and lack of funding were among the most predominant and common barriers experienced by healthcare personnel (Cooper et al., 2016; Gwernan‐Jones et al., 2020; Monthaisong, 2018; Smythe et al., 2017).

Nurses’ experience of care was also found to be influenced by the staff's working condition. For example, in the UK, researchers found poor staff working conditions, undertrained nurses and a negative image of the work setting had a negative impact, but despite these factors, nurses were compassionate towards dementia patients (Digby et al., 2018; Kupeli et al., 2016). A Swedish study found that nurses struggled to perform person‐centred care for dementia patients, and most did not hold positive attitudes towards dementia patients (Ericson‐Lidman et al., 2014); this is also confirmed by focused groups studied in Germany and Austria (Pinkert et al., 2018). While in China, authors claimed that nurses caring for people with dementia in community care settings failed to apply person‐centred care. In Australia, nurses argued that non‐pharmacological interventions were not the nurses’ role, claiming that they always worked under pressure (Digby et al., 2018; Ervin et al., 2014; Shannon et al., 2018). Therefore, nurse–patient communication and a conducive environment played an important role in changing the nurses’ perceptions towards patients with dementia (Beck et al., 2017; Krupic et al., 2016; Rosendahl et al., 2016).

In contrast, studies conducted in acute care settings revealed poor quality of services and a lack of knowledge about dementia. Issues like burnout and high‐stress levels were common in patients. Nurses’ burnout and lack of essential knowledge of dementia incapacitate the delivery of quality services. In Japan, nurses within psychiatry wards who were working with aggressive dementia patients had high‐stress levels and experienced burnout (Yada et al., 2014). However, if workplace environment stressors were reduced for nurses, the cognitive health of patients with dementia improved (Yada et al., 2014). In the UK, work pressures on nurses were influenced by different perceptions of patients with dementia (Lillekroken et al., 2015; Midtbust et al., 2018a); some indicted compassion and love while others experienced psychological stress (McPherson et al., 2016; Monthaisong, 2018; Yada et al., 2014). Thus, the work environment varied for nurses in different settings. A study evaluating dementia care experience from the perspective of nurses reported that inadequate staffing, along with few educational training opportunities, undermines the quality of care (Yous et al., 2019).

Job satisfaction was also linked to dementia care experience in both acute care settings and community care settings. Community care setting nurses from the Netherlands found that the level of nurses’ job satisfaction, motivation and social support were different among those taking care of dementia patients (Adams et al., 2017). The most significant factor that was correlated with higher job satisfaction and motivation was social support (Adams et al., 2017). Inadequate nursing support leads to low job satisfaction in nurses taking care of dementia patients (Adams et al., 2017; Digby et al., 2017). In both acute and community care settings, nurses may have to strive to understand the conditions of dementia patients and even struggle to adapt to the new cultural norms of offering quality care to these patients (Egede‐Nissen et al., 2017; Evripidou et al., 2019). The nurses’ perceptions towards dementia patients may vary depending on the severity of the dementia and even based on the patient's religion, ethnicity and gender (Tomlinson & Stott, 2015).

#### 3.4.3 Theme 3: Dementia nursing care across health regions

In the UK, the six qualitative studies mostly focused on investigating nurses’ perceptions of dementia patients in both acute and community care settings (De Witt et al., 2017; Kupeli et al., 2016; Lee et al., 2017; McPherson et al., 2016; Ross et al., 2015; Smythe et al., 2017). Despite the nurses acknowledging the importance of end‐of‐life care, they struggled to administer good end‐of‐life care to dementia patients (Lee et al., 2017; McPherson et al., 2016). In Norway, six qualitative studies were conducted in community care settings to investigate nurses’ perceptions towards dementia patients and the nurses’ caregiving (De Witt et al., 2017; Hansen et al., 2017; Jakobsen & Sørlie, 2016; Lillekroken et al., 2015; Skomakerstuen Ødbehr et al., 2015; Solli et al., 2015). Work pressure and nurses’ burnout can be perceived by nurses to be the most significant reasons behind the mismanagement of dementia‐afflicted patients (Chaudhury et al., 2017; Hunter et al., 2017; Midtbust et al., 2018b). In addition, these nurses also expressed that they felt that it is regular practice, which can assist in enhancing their knowledge together with educational interventions. This can further contribute to encouraging a positive attitude with respect to communication and personal interaction (de Witt & Ploeg, 2016; Eritz et al., 2016; Machiels et al., 2017). In the United States, four quantitative studies were conducted in both settings (Brody et al., 2016; Daniel et al., 2014; Karlin et al., 2017; Unroe et al., 2015), along with one mixed‐method study (Sharpp & Young, 2016) in an acute care setting (dementia wards at the hospital), to gather a clear inference of the nurses’ knowledge; this was done by investigating the nurses’ attitudes towards dementia patients and towards dementia care. The diversities in the practice and skill of the nurses led to an increase in the level of risks over time (de Witt & Ploeg, 2016; Unroe et al., 2015).

A few mixed‐methods studies have been conducted within community and acute care settings to investigate nurses’ knowledge of providing the required care to dementia‐affected people (Cooper et al., 2016; Naughton et al., 2016; Smythe et al., 2014). However, considering the concept of communication, it can be inferred that a lack of empathy along with the aggressive behaviour of dementia patients tends to hamper the proper decision‐making of nurses. One qualitative and one quantitative study in community care contributed to inferring the importance of non‐verbal communication in decision‐making (Krumm et al., 2014; Krupic et al., 2016; Kuehlmeyer et al., 2015; Saxell et al., 2019). A control trial study in Switzerland community care focused on nurses’ knowledge (Kohler et al., 2016) and concluded that educational programmes improve the quality of care provided and could further reduce urinary incontinence issues in dementia patients. In South‐East Asia, a mixed‐method study was conducted in an acute care setting (medical ward) in South Korea (Kang et al., 2017), and there was a review that included South Korean nurses (Evripidou et al., 2019). It was declared that educational strategies alter self‐confidence, which further leads towards improving the assessment of dementia patients (Kang et al., 2017). In Palestine, within community care settings, a significant variation in attitudes towards the autonomy and dignity of patients with dementia has been noted among 196 nurses (Bentwich et al., 2017). The researchers suggested that the nurses lacked positive perceptions of dementia patients, which further resulted in a failure to maintain autonomy and/or dignity in their care. This explains how nursing competency and the care environment can contribute to the dignity and quality of life of dementia patients (Jenkins, 2016).

## 4 DISCUSSION

The main objective of the current study was to synthesise a comprehensive body of evidence about nurses’ knowledge, attitudes and practices towards dementia care. Another objective was to explore experience of care in acute care hospitals and community care settings and evaluate experience by different regions across the world. A total of 72 articles were identified from the literature search, and these were classified into three themes. The majority of the reviews included qualitative or quantitative papers, but there were a few trials.

The first theme reported on the knowledge, perceptions and attitudes of nurses towards dementia care. A total of 72 studies explored this topic. The studies revealed that most nurses had basic knowledge about dementia. However, deficiencies in specific knowledge, such as onset to end‐of‐life care and pain management, were frequently reported (Beck et al., 2017; Burns & McIlfatrick, 2015a; Deasey et al., 2014; Naughton et al., 2016; Nilsson et al., 2016; Unroe et al., 2015). Handling the aggressive behaviour of dementia patients was challenging for many nurses, and they reported poor therapeutic relationships with staff because of this issue. The studies also revealed the role of inadequate training and the absence of lessons on dementia management in the nursing curriculum. Most of the studies were of a high or good quality. However, the gaps that remained in addressing the first theme were that most of the studies were done using convenience sampling or purposive sampling methods. In addition, for end‐of‐life care, it remains to be seen how new service initiatives may help nurses support patients during end‐of‐life care. In the case of studies that reported concerns related to the management of aggressive behaviour, the findings were restricted to studies done in two or three inpatient units. Thus, recruiting samples from a broader range of services can help to generalize these findings. Despite this limitation, the findings are consistent with a recent research evidence, who argue that care delivery for dementia is challenging for nurses because they often navigate through patients’ various feelings and emotions (Yous et al., 2019). They often experienced the ethical dilemma of feelings of anger when met patients who shows aggressive behaviour (Yous et al., 2019).

In response to the low knowledge and skill level of nurses, many countries have implemented educational programmes to increase competency in dementia care for health staff. These studies highlighted the benefits of educational programmes in improving nurses’ confidence, knowledge and attitudes towards dementia patients. This signifies the role of education in boosting confidence in care delivery for this health issue (De Witt et al., 2017; Eritz et al., 2016; Schindel Martin et al., 2016). The evidence‐based findings from the current review study give a clear indication that the creation of awareness about dementia and experience in dealing with dementia patients improves both knowledge and the general skill sets that are relevant to dementia patients (Wang, Xiao, et al., 2017; Wang, He, et al., 2017). Therefore, educational interventions and training programmes are an important resource for future improvement of the competency levels of dementia in nursing care. This helps to enhance nurses’ ability to manage pain issues that are often not verbally expressed by patients. However, there were some quantitative surveys that used questionnaires lacking content validity. This limitation is addressed later.

The second theme was concerned with evaluating nurses’ experiences in acute and community care settings. A wide diversity in practice was found. However, each of the settings had different limitations, and different actions were taken to enhance care. For instance, continuous professional development initiatives were evident in many community care settings (Witt & Ploeg, 2016; Egede‐Nissen et al., 2017; Eritz et al., 2016). In contrast, there were many studies that were affected by a poor leadership style. A UK‐based study focused on the fact that most nursing managers lacked proper knowledge of managing dementia patients; they even possessed negative attitudes towards planning for advanced care of dementia patients (Beck et al., 2017). A researchers also claimed that leadership style influences the attitudes towards caring, thus negatively affecting the nurses’ working atmosphere, ultimately creating a somewhat negative impact on their work experiences (Jakobsen & Sørlie, 2016). Thus, studies reported under this theme highlight the role of working environment and leadership style in dementia care. Job satisfaction and other forms of social motivation also greatly influenced the nature of the services offered by nurses to dementia patients. However, the gap that remains in addressing this theme is that the theme does discuss the role of community services in improving the care experience of these people. Thus, future research could evaluate how nurses’ knowledge and access to community services can broaden their understanding of safety (Kable et al., 2015). This will help in finding out how a care transition takes place from acute care to community care settings.

The third theme was related to experience of dementia care in different regions across the world. It has been found that the issue of burnout and work pressure is not limited to one setting. Studies done in Europe, the United States and Eastern Mediterranean countries reported these issues. Poor patient autonomy and violation of dignity in care were a major concern. For this reason, nurses are recommended to engage family members in the care to ensure that information about the patient's premorbid function and their likes/dislikes could be obtained (Sagbakken et al., 2017).

Overall, the present review contributes to more knowledge about the current knowledge and attitude of nurses and how their knowledge, attitudes and perceptions is enhanced or reduced by different factors in care; it gives guidance about the areas that need more work.

### 4.1 Strengths and weaknesses of the review

This review mainly aims to uncover the knowledge, attitudes and practices of nurses in dementia care. The strength of the review is that it includes 72 articles from diverse settings. In addition, not limiting the research to any specific research design was also a strength because it ensured that diverse types of papers were reviewed. However, one major limitation of the review is that it lacked the inclusion of many randomized control trials. Because randomized control trial studies come under top quality in evidence hierarchy, including a few randomized control trial studies were important. The lack of these studies leaves a gap in the integrated review process. Second, the review focused on the quality of each article. Hence, most of the studies were high‐quality or good‐quality papers, which were mostly evaluated using the JBI critical appraisal tools. The strength of the review is that no studies were found that had a quality appraisal score of less than 50%.

### 4.2 Limitations of the studies

The main limitation of this study is it has been conducted by one researcher; however, all decisions and findings were discussed in supervisory meetings in order to reduce researcher's bias and maintain transparent findings and conclusion. Also, unequal data were found across the health regions and scarcity of literature available in most developing countries in Eastern Mediterranean and Asian region in particular.

## 5 IMPLICATIONS FOR POLICY OR PRACTICE CHANGE

To achieve best nursing practices and enhance dementia nursing care, researchers across the globe have acknowledged the importance of exploring and investigating nurses’ knowledge, attitudes and perceptions towards people with dementia. Researchers have used different research methodologies to achieve this aim; however, there is an absence of studies in particular areas, such as in the Eastern Mediterranean and South‐East Asia regions.

The current review's findings indicate that improving the practical experience of nurses enhances their knowledge of dementia, thereby leading to an improved quality of care. It is found that skill‐based training further increases knowledge by improving the communication on and problem‐solving skills of staff members working with dementia patients in acute settings. Also, a patient's inability to verbally communicate with nurses about their problems, including pain, makes them vulnerable and highly dependent on their caregivers. To improve pain relief during end‐of‐life care, professional experience, an understanding of the patient's background and the use of pain assessment tools are required. Developing a psychometric assessment tool for nurses to determine patients’ requirements, such as pain assessment and the activities of daily living, can contribute to enhancing the person with dementia hospitalization experience. Therefore, it can be concluded that the healthcare settings and culture, along with the knowledge of the nurses, their attitudes and their perceptions, can have a tremendous impact on dementia‐affected individuals.

This review also found that appropriate communication further plays a significant role in the development of a healthy environment between dementia patients and healthcare providers. The establishment of verbal and non‐verbal communication protocols in nursing care for dementia is vital in providing dementia nursing care. In addition, nurse–physician relationships are vital for facilitating communication at the time of deciding the care plan of dementia patients. Also, the work environment and care settings play an important part in the nurses’ perceptions, along with dementia care delivery. The burnout of nurses and lack of essential knowledge on dementia among them further incapacitates the delivery of quality services within acute or community care settings. If the workplace environment stressors were to be reduced for nurses, the cognitive health of patients with dementia would most likely be improved. Job satisfaction and other forms of social motivation greatly influence the nature of the services offered by nurses to dementia patients. Work pressure and inadequate time and availability of training programmes are noted to be the foremost barriers to educational improvement strategies as perceived by nursing professionals. Work pressure along with nurses’ burnout can also be perceived as being the most significant reasons behind the mismanagement of dementia‐afflicted patients.

Therefore, providing healthcare educational programmes and then setting up continuous professional development initiatives are the key principles of person‐centred care approaches. The lack of educational opportunities, limited access to training, staff shortages, lack of management support, time constraints and lack of funding are the factors to be addressed for improving dementia care. Additionally, leadership style influences the attitudes towards caring, thus negatively affecting the nurses’ working atmosphere, which ultimately creates a somewhat negative impact on their work experiences.

## 6 CONCLUSION

Overall, the present review contributes to the understanding of the current knowledge and attitude of nurses and how their knowledge, attitudes and perceptions are enhanced or reduced by different factors in care; it gives guidance about the areas that need more work.

In particular, the studies found that having samples from a broader range of services can help researchers draw a more generalized understanding of how nurses may navigate through the ethical dilemmas of dealing with patients’ various emotions and aggressive behaviours. Moreover, the role of working environment and leadership style in dementia care is vital for job satisfaction, which influences the care offered by nurses to people with dementia. Lastly, the issue of burnout and work pressure resulting from poor patient autonomy and violation of dignity in care is a major concern that affects nurses in all regions of the world.

Thus, future research needs to focus on educational interventions and training programmes, as these are an essential component in raising nurses’ competency levels in managing patients’ pain issues. In addition, evaluating nurses’ knowledge and access to community services can broaden their understanding of safety and help in transitioning patients from acute care to community care settings. Finally, nurses are advised to engage family members to ensure that the patient's care includes adjustments made for premorbid function and personal preferences.

This review mainly aims to synthesise the knowledge, attitudes and practices of nurses in dementia care. The strength of the review is that it includes 72 articles from diverse countries. In addition, not limiting the research to any specific research design was also a strength because it ensured that diverse types of studies were reviewed. This review focused on the quality of each article. Hence, most of the studies were high‐quality or good‐quality papers, which were mostly evaluated using the Joanna Briggs Institute critical appraisal tools. The strength of the review is that no studies were found that had a quality appraisal score of less than 50%.

It is everyone's right to have positive experiences and receive the right support when admitted to an acute or a community care facility. This study contributed to the current body of knowledge on the dementia nursing care and suggested possible areas that need more development and concerns to enhance the nursing care for people with dementia, which, in turn, can influence their well‐being.

## CONFLICT OF INTEREST

The authors have no conflicts of interest that are directly relevant to the content of this review.

## ETHICAL APPROVAL

Ethical approval was not required.

## Data Availability

Due to the nature of this research, integrative literature review, most studies and research papers are available within the databases mentioned in the methods section; some studies are opened accessed and other available according to institutions. For more information regarding the data availability, please contact the author.
